# Experimental Study on Damage Effect of Mid-Infrared Pulsed Laser on Charge Coupled Device (CCD) and HgCgTe Detectors

**DOI:** 10.3390/s24134380

**Published:** 2024-07-05

**Authors:** Yang Liu, Feng Zhou, Yunzhe Wang, Yin Zhang, Yunfeng Zhang, Hanyu Zheng, Junfeng Shao

**Affiliations:** 1 Changchun Institute of Optics, Fine Mechanics and Physics, Chinese Academy of Sciences, Changchun 130033, China; liuyangdk@ciomp.ac.cn (Y.L.); zhangyunfeng@ciomp.ac.cn (Y.Z.); zhenghanyu22@mails.ucas.ac.cn (H.Z.); 13159754836@163.com (J.S.); 2 Beijing Blue Sky Innovation Center for Frontier Science, Beijing 100049, China; zhougj776@sohu.com; 3 University of Chinese Academy of Sciences, Beijing 100049, China

**Keywords:** mid-infrared pulsed laser, CCD detector, HgCdTe detector, damage threshold, out-band

## Abstract

As the weak link in electro-optical imaging systems, photodetectors have always faced the threat of laser damage. In this paper, we experimentally investigated the damage mechanism of the photodetector induced by the out-of-band laser. The damage thresholds of the mid-infrared pulsed laser for Charge Coupled Device (CCD) and HgCdTe detectors were determined through damage experiments. The analysis of the damage phenomena and data for both CCD and HgCdTe detectors clearly demonstrated that out-of-band mid-infrared pulsed lasers could entirely incapacitate CCD and HgCdTe detectors. Our analysis of the damage process and data revealed that the primary mechanism of damage to CCD and HgCdTe detectors by mid-infrared pulsed lasers was primarily thermal. This study serves as a reference for further research on the mid-infrared pulsed laser damage mechanisms of CCD and HgCdTe detectors, as well as for laser protection and performance optimization in imaging systems.

## 1. Introduction

When the photodetector is irradiated by a significantly stronger laser than the normal signal light, it readily saturates, diminishing its performance, and may experience temporary or even permanent failure [1,2,3,4]. The absorption of the photodetector material by the “in-band” laser is primarily intrinsic, with an absorption coefficient ranging from 10^5^ cm^−1^ to 10^6^ cm^−1^. However, the absorption coefficient of the photodetector for the “out-band” laser is considerably lower, approximately four to five orders of magnitude lower than that for the “in-band” laser [5]. Consequently, there has been limited research on the damage caused to photodetectors by “out of band” lasers. With the advancement of high-energy laser technology, researchers have discovered that “out of band” lasers can induce interference and damage to detectors when the energy levels are sufficiently high [6,7,8].

Pei Gee Chua et al. [9] from Tokyo University of Agriculture and Technology conducted experiments wherein a CCD was irradiated with a 1550 nm fiber continuous-wave laser (the cutoff wavelength of the photodetector being 1100 nm) and observed a robust response of the CCD to the 1550 nm CW laser. The experimental data indicated that the CCD began to respond when the energy density reached only 300 W/cm^2^, a power density significantly lower than that required for two-photon absorption of silicon materials, which typically involves megawatts per square centimeter. Matthias Loch and Erik Bodegom et al. [10,11] investigated the response of CCDs to “out of band” (1200–1600 nm) lasers. The experimental results revealed that CCDs exhibited a response to out-of-band lasers, with the response rate decreasing as the wavelength increased and quantum efficiency increasing with temperature. Hust’s Professor Cai Hu researched the thermal stress damage, evaporation wave stress, and detonation wave stress of Hg_0_._8_Cd_0_._2_Te crystal materials under a 10.6 μm TEA CO_2_ pulsed laser and pointed out that thermal effects were the main cause of damage to the wafers [12]. He further explained that the thermal stress during laser irradiation led to the formation of network cracks on the material surface due to different thermal expansions caused by thickness inconsistencies during solidification. Wang Fei and others conducted research on PV-type HgCdTe detectors using continuous and quasi-continuous lasers at 1064 nm and 1319 nm wavelengths, discovering that the precipitation or diffusion of Hg atoms and the degradation of PN junctions were significant reasons for the decreased signal light response of the detectors [13]. In 2009, Li Li from the National University of Defense Technology used a combination of 1.319 μm and 10.6 μm dual-band lasers to irradiate PC-type HgCdTe detectors, finding that background laser irradiation within the band could increase the absorption coefficient of out-of-band lasers, and the voltage response of the detector to out-of-band laser irradiation also exhibited a saturation effect [14]. Zhang Yue and others conducted research on the damage mechanism of InSb infrared detectors using a 10.6 μm laser, discovering that the damage threshold for indium columns in the detector was about 16.3 J/cm^2^, higher than the melting damage threshold of InSb material at 1.3 J/cm^2^, and that the melting damage threshold of the material remained relatively constant for pulse widths shorter than 10 ns [15]. Hu Weimin and others established a one-dimensional self-consistent model to simulate the carrier density and lattice temperature of HgCdTe crystal materials under laser irradiation. They studied the damage thresholds of HgCdTe materials using 2.85 μm mid-infrared lasers with different pulse widths (30 ps to 10 ns), finding that when the pulse width was less than 3 ns, the damage threshold of the detector was around 200 mJ/m^2^, lower than the 474 mJ/cm^2^ damage threshold at a 10 ns pulse width [16]. The analysis suggested that the picosecond pulses, due to their duration being close to the electron-lattice energy transfer time, caused electrical surges and shock stress, leading to optical breakdown on the material surface. In 2022, Wang Xi and others conducted damage experiments and simulations on PbS detectors using repetitive 2.79 μm mid-infrared lasers [17]. The results showed that soft damage to the detectors was caused by the reduction in the response voltage due to mid-infrared laser irradiation. The laser irradiation caused thermal decomposition of PbS, producing yellow precipitates of PbO, which was the core reason for the permanent damage to the detectors. Simulation calculations determined that the damage threshold of single-pulse laser irradiation on PbS detectors was 13.03 J/cm^2^. The experimental results revealed that CCD and HgCdTe exhibited a response to out-of-band lasers, with the response rate decreasing as the wavelength increased and quantum efficiency increasing with temperature. Nevertheless, further research is required to investigate the damage threshold and mechanism of out-of-band lasers on detectors.

**Figure 1:**
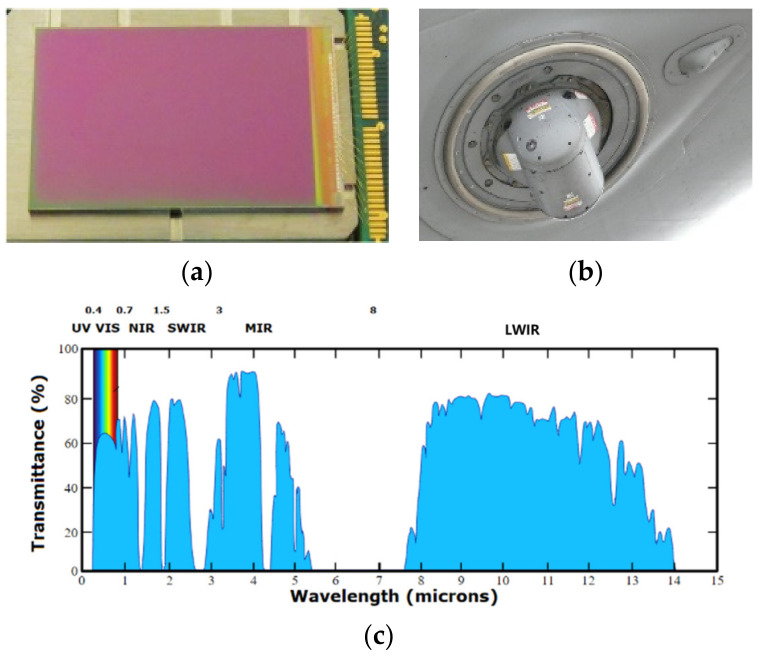
Applications of mid-infrared band. (**a**) HgCdTe detector chip. (**b**) Application of the detector in photoelectric load. (**c**) The atmospheric window.

As shown as in Figure 1, mid-infrared lasers, situated within the eye-safe spectrum, play crucial roles in various applications such as lidar, atmospheric analysis and detection, medical diagnoses, material processing, battlefield communications, and optoelectronic countermeasures [18,19]. For instance, employing lidar in the 2 μm band enables direct measurement of wind speed, a crucial parameter for weather forecasting, typhoon tracking, and aviation safety. As illustrated in Figure 1, the 3~5 μm band represents the atmospheric window. Many laser-directed energy infrared jamming systems, including DIRCM systems, employ mid-infrared lasers, while the fairings of most infrared-guided missiles feature 3~12 μm anti-reflection coatings. It is evident that in the foreseeable future, the mid-infrared band will encompass vast application domains in both military and civilian sectors [20,21,22,23].

The current research on the damage inflicted by “out-band” lasers on detectors predominantly concentrates on infrared detectors and CCD detectors. Certain researchers have conducted experiments investigating the detrimental effects of continuous-wave (CW) lasers in visible light and near-infrared bands on detectors [24,25]. The findings indicated that the mechanism by which “out of band” CW lasers impaired photodetectors was due to thermal accumulation. Limited research has been conducted on the damage inflicted on detectors utilizing mid-infrared lasers as light sources, with no reports available regarding the damage of CCDs by mid-infrared pulsed lasers. However, there was a lack of systematic research on the damage mechanism, process, and effects of mid-infrared pulsed lasers on CCDs. This paper conducted laser damage experiments using a 3.8 μm mid-infrared pulsed laser on CCDs and HgCdTe detectors, obtaining threshold data for damage caused by mid-infrared pulsed lasers on both types of detectors. The mechanism of laser-induced damage to CCDs and HgCdTe detectors was explored. The damage mechanism of mid-infrared pulsed lasers on CCDs and HgCdTe detectors was elucidated through comparative analysis of damage processes and morphologies.

## 2. Theoretical Model of Laser Irradiation Photodetector

### 2.1. CCD Detector Laser Irradiation Model

In analyzing the thermal conduction process within CCDs, the thermal conduction differential equation was employed to compute the temperature distribution across the thermally conductive object. In accordance with the principle of energy conservation, the general form of the three-dimensional unsteady thermal conduction differential equation can be expressed as

(1) $$\rho c\frac{\partial T}{\partial t}=\frac{1}{r}\frac{\partial }{\partial r}(kr\frac{\partial T}{\partial r})+\frac{1}{r^{2}}\frac{\partial }{\partial \varphi }(k\frac{\partial T}{\partial \varphi })+\frac{\partial }{\partial z}(k\frac{\partial T}{\partial z})+Q$$

Here, *k* represents the thermal conductivity of the material, *ρ* denotes its density, *c* stands for the heat capacity, *T* signifies the temperature, and *t* represents time. The left side of the equation represents the change in thermodynamic energy of the microelement per unit time, known as the unsteady term. On the right side of the equation, the sum of the first three terms represents the energy increment of the microelement per unit time due to thermal conductivity across interfaces, known as the diffusion term. The final term, represented by *Q*, denotes the heat source term.

The heat source term *Q* for the pulsed laser can be formulated as follows:

(2) Q(r,z,t)=I(1−R)αexp⁡(−αz)

Here, *I* represents the peak power density of the pulsed laser, while *R* denotes the reflectivity.

The temperature increased rapidly near the area irradiated by the laser, whereas the temperature within the CCD changed gradually, leading to an uneven temperature distribution across the detector and consequent thermal stress. Because of the disparate thermal expansion coefficients among the materials comprising the layers of the CCD, temperature elevation induced varying degrees of deformation in these layers, thereby giving rise to thermal stress. The stress field was linked to the temperature field in the following manner:

(3) $$\sigma_{ir}(r,z)=\frac{\beta_{i}E_{i}}{1-\gamma_{i}}\left[\frac{1}{R_{0}^{2}}\int_{0}^{R_{0}}\;T_{i}rdr-\frac{1}{r^{2}}\int_{0}^{r}\;T_{i}rdr\right]$$

(4) $$\sigma_{i\theta }(r,z)=\frac{\beta_{i}E_{i}}{1-\gamma_{i}}\left[\frac{1}{R_{0}^{2}}\int_{0}^{R_{0}}\;T_{i}rdr+\frac{1}{r^{2}}\int_{0}^{r}\;T_{i}rdr-T_{i}\right]$$

(5) $$\sigma_{iz}(r,z)=\frac{\beta_{i}E_{i}}{1-\gamma_{i}}\left[\frac{1}{R_{0}^{2}}\int_{0}^{R_{0}}\;T_{i}rdr-T_{i}\right]$$

**Figure 2:**
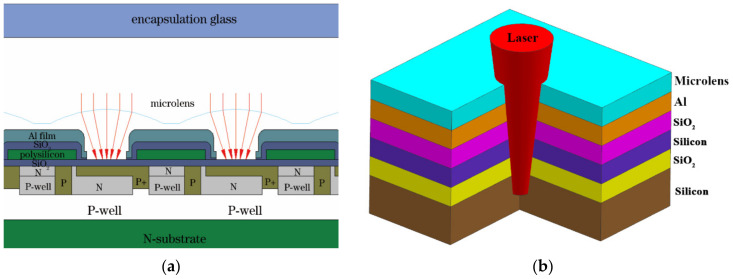
Fundamental structure diagram of typical CCD pixel. (**a**) Typical CCD detector microstructure diagram. (**b**) Diagram of laser irradiation on CCD.

Here, *σ_ir_*(*r*,*z*), *σ_iθ_*(*r*,*z*), and *σ_iz_*(*r*,*z*) represent the radial, hoop, and axial thermal stresses of the *i*th layer, respectively. The variables r and z denote the radial and incident directions, respectively. *β_i_* denotes the thermal expansion coefficient of the *i*th layer material, Ei signifies the Young’s modulus of the *i*th layer material, *γ_i_* represents the Poisson’s ratio of the *i*th layer material, and R_0_ denotes the radius of the cylindrical coordinate system. The temperature and stress distributions within the CCD model can be determined by solving Equations (3) through (5). Materials like polycrystalline Si typically exhibit thermal damage phenomena including melting, evaporation, cracking, and thermal decomposition when the irradiation time exceeds 10^−7^ s, attributed to thermal stress. Owing to the short electro-optical coupling time of Si-based materials, the material’s surface temperature rapidly escalates upon laser irradiation, ultimately surpassing its melting point at localized regions, thereby leading to thermal melting damage. The structure and simplified model of the CCD are illustrated in Figure 2. Model calculations indicated that the microlens reached its melting point first, whereas the melting point of the Si-based bottom layer was relatively high.

### 2.2. HgCdTe Detector Laser Irradiation Model

With funding from the US Navy, F Bartoli systematically conducted research on the damage mechanisms of multi-wavelength lasers on diverse infrared detector materials. Moreover, a thermal damage model for CO_2_ lasers on HgCdTe materials was developed. Between 1975 and 1977, F Bartoli [26,27,28,29] developed a thermal damage mechanism model adaptable to various parameters including pulse width and material thickness. Additionally, systematic investigations were conducted on the damage effects of lasers with diverse pulse widths and wavelengths on various infrared materials, yielding widely recognized damage effect data. The physical model depicting the interaction between the laser and the material encompassed the heat absorption and heat conduction processes of the material under laser irradiation, formulated as Equation (1), where *T* is the temperature value of the crystal at time *t*, *k* is the thermal conductivity of the material, *ρ* is the density, *c* is the specific heat capacity, *α* is the absorption coefficient, and *I*_0_ is the irradiation laser power density.

Utilizing the aforementioned differential equation, the calculation formula for the thermal damage threshold *E*_0_ induced by a Gaussian beam on HgCdTe material could be derived as follows:

(6) $$E_{0}=E_{\Delta T}\left(1+\frac{k\tau \alpha \pi^{\frac{1}{2}}}{a_{0}{tan}^{-1}\left[(4k\tau /a_{0}^{2}{)}^{\frac{1}{2}}\right]}\right)$$

where *E*_Δ*T*_ is

(7) $$E_{\Delta T}=\frac{\Delta T_{th}\rho c}{(1-R)\alpha }$$

where *k* is the thermal diffusivity, Δ*T_th_* is the difference between the temperature from the ambient temperature to the thermal melting temperature of 993 K, *R* is the reflectance of the photosensitive surface, *c* is the specific heat capacity, *ρ* is the material density, *τ* is the laser pulse width, *α* is the material absorption coefficient, and *a*_0_ is the Gaussian spot radius.

Based on the model, the preliminary estimation of the damage threshold of mid-infrared pulsed laser on HgCdTe material was conducted. While some parameters adhered to those utilized by Bartoli in theoretical analysis, others were adjusted to fit the conditions of the medium-wave laser. Specific parameters of HgCdTe are detailed in Table 1.

## 3. Experimental Research on Mid-Infrared Pulsed Laser Damage HgCdTe Detectors

### 3.1. Experiments on HgCdTe Detectors Damaged by Mid-Infrared Pulsed Laser

**Figure 3:**
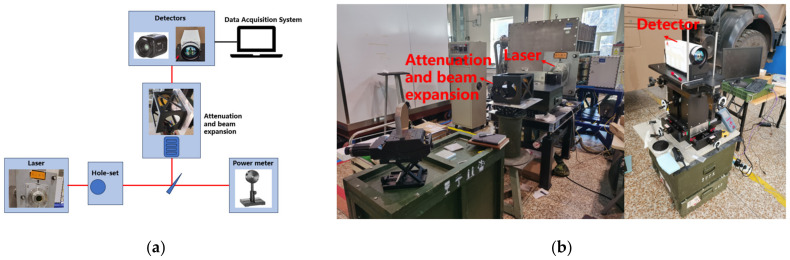
Laser damage effect experimental platform. (**a**) Detector damage experimental plan. (**b**) Detector damage experimental platform.

The experimental setup is depicted in Table 2, utilizing a high-energy DF laser as the primary light source. In accordance with the energy density formula, the damage threshold exhibited a quadratic relationship with the spot radius. For instance, with a 100 mm focal length lens, under parallel laser conditions, the spot size at the focal plane is typically within microns. Any error in the beam diameter will result in a squared amplification effect on the final damage threshold. Therefore, precise measurement of the spot size is crucial for the experiment’s success. Conventional knife-edge methods are impractical under the irradiation of high-energy light spots, resulting in distortion of the light spot to some extent even after attenuation by a small number of superimposed attenuators. However, this distortion has minimal impact on the spot area. Thus, the attenuated HgCdTe detector’s direct observation method was employed for spot size determination. The beam’s divergence angle was initially compressed via a hole-set, following which it was split by a wedge mirror. The resulting weak light was observed directly using an HgCdTe camera. The focal plane’s position was adjusted using a precise displacement platform (Platform X), and an image extraction algorithm was utilized for spot size calculation. Ensuring the repeatability of the focal plane position, both the photodetector under test and the spot monitoring camera were mounted on the same displacement platform (Platform X). The experimental platform layout is depicted Figure 3.

The HgCdTe detector utilized in the experiment possessed pixel dimensions of 30 μm × 30 μm, while the supporting lens featured a focal length of 100 mm and an aperture of 50 mm. Calculations based on parallel light incidence yielded a diffraction-limited spot size of 20 μm, whereas a spot diameter of 1000 μm was computed considering a non-parallel light incidence with a divergence angle of 10 mrad. The latter, larger beam diameter proved detrimental to the experiment, favoring the occurrence of interference, point, line, and blinding thresholds. Thus, employing parallel light incidence facilitated easier acquisition of such thresholds. Calculations based on parallel light incidence yielded a diffraction-limited spot size of 20 μm, whereas a spot diameter of 1000 μm was computed considering a non-parallel light incidence with a divergence angle of 10 mrad. The larger beam diameter resulting from non-parallel light incidence proved detrimental to the experiment, favoring the occurrence of interference, point, line, and blinding thresholds. Thus, employing parallel light incidence facilitated easier acquisition of such thresholds. The laser spot, resembling the TEM_20_ mode, underwent partial energy interception and divergence angle compression through a diaphragm, followed by utilization of a medium-wave 10X beam expander to achieve parallel light for experimentation. Employing a 1-on-1 mode, laser energy was adjusted using the attenuation module, culminating in the determination of the laser damage threshold within the specified wavelength range.

### 3.2. Spot Diameter Measurement to Photodetector

The experimental approach for measuring the spot diameter involved direct measurement of both the test object and the optical system. Additionally, statistical analysis was conducted, leveraging the second-order moment theory of laser transmission characteristics.

The spot diameter was determined utilizing the second-order moment (or 4σ), wherein the energy within the 4σ diameter encompassed 86.5% of the total laser energy. Hence, the spot diameter was established as four times the standard deviation of the energy distribution, independently computed in the X and Y directions.

(8) $$d_{\sigma_{x}}=4\sigma_{x}$$

(9) $$d_{\sigma y}=4\sigma_{y}$$

where *d_σ_* is the diameter of the spot, and *σ_i_* (i = x, y) is the standard deviation of the spot energy distribution in the x and y directions.

The formula for computing the standard deviation of the laser energy distribution was expressed as follows:

(10) $$\sigma_{x}^{2}=\frac{\sum_{x}\;\sum_{y}\;(x-\bar{x}{)}^{2}\cdot Z(x,y)}{\sum_{x}\;\sum_{y}\;Z(x,y)}$$

(11) $$\sigma_{y}^{2}=\frac{\sum_{x}\;\sum_{y}\;(y-\bar{y}{)}^{2}\cdot Z(x,y)}{\sum_{x}\;\sum_{y}\;Z(x,y)}$$

where Z(x,y) is the pixel size. X and Y are the coordinates of the spot center. Its calculation formula was

(12) $$x_{centroid}=\frac{\sum (X\times Z)}{\Sigma Z}$$

(13) $$y_{centroid}=\frac{\sum (Y\times Z)}{\Sigma Z}$$

**Figure 4:**
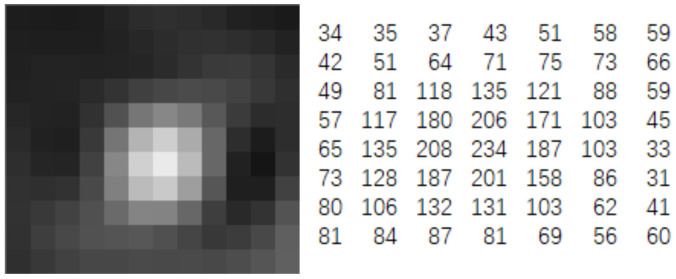
Laser spot imaging with mid-infrared detector.

The gray value distribution matrix obtained by the laser in the mid-infrared camera is shown in Figure 4.

### 3.3. Experimental Results of Mid-Infrared Pulsed Laser Damage to HgCdTe Photodetector

**Figure 5:**
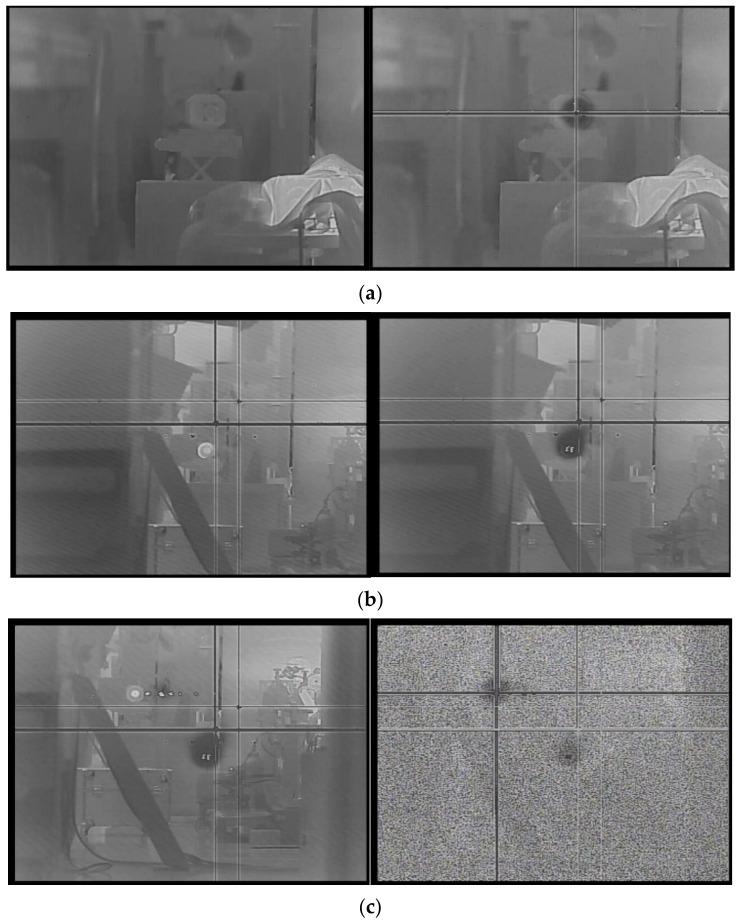
Experiment on HgCdTe detector irradiated by mid-infrared pulsed laser. Response of the photodetector during irradiation (**left**). Imaging effect of detector after damage (**right**). (**a**) Point damage. (**b**) Line damage. (**c**) Complete damage.

According to the experimental findings, an escalation in energy density precipitated the gradual emergence of three phenomena in the HgCdTe photodetector: point damage, line damage, and eventual complete damage (see Figure 5). Specifically, in the 1-on-1 mode, point damage initiated at an energy density of 0.412 J/cm^2^, followed by the onset of line damage at 0.551 J/cm^2^, culminating in complete photodetector failure at an energy density of 0.953 J/cm^2^.

The experiment yielded a damage threshold ranging from 0.41 to 0.95 J/cm^2^, aligning with previous theoretical calculations. The slight variance between the two can be attributed to the Gaussian energy distribution of the light spot in this model, which differed slightly from the TEM20 distribution observed in the experiment.

## 4. Experiment of Mid-Infrared Pulsed Laser Damage to CCD Detectors

### 4.1. Transmittance Measurement of Mid-Infrared Laser to CCD Packaging Glass

**Figure 6:**
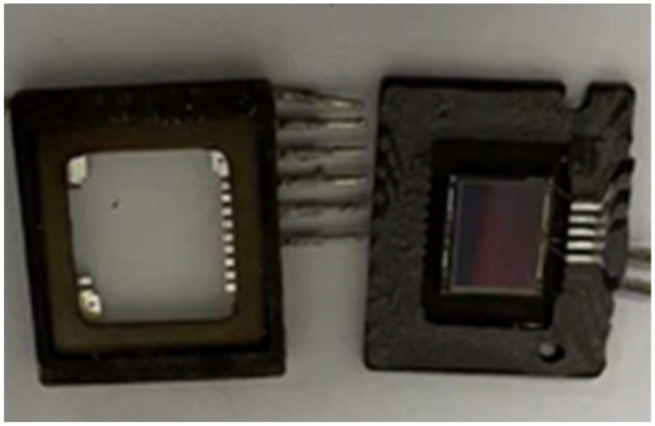
CCD packaging glass (**left**) and CCD chip (**right**).

**Figure 7:**
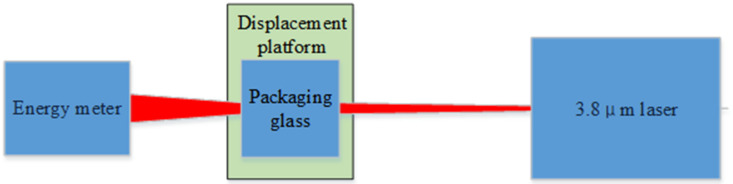
Schematic diagram of transmittance test of CCD packaging glass.

**Figure 8:**
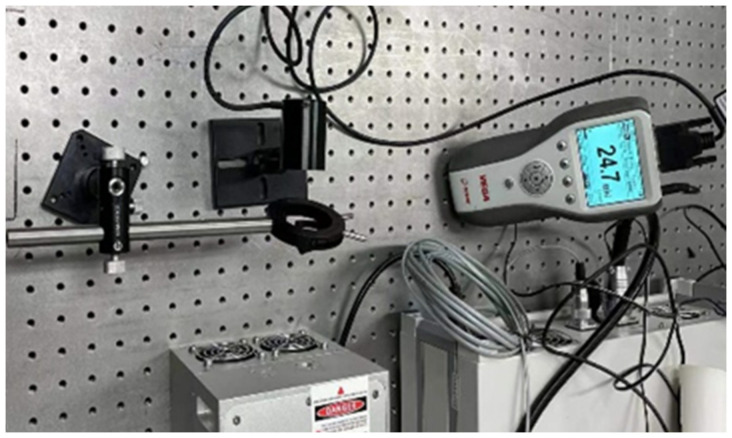
CCD package glass transmittance measurement experimental platform.

The visible light CCD camera consisted of packaging glass, a CCD chip, and a back-end circuit (see Figure 6). The packaging glass primarily served to safeguard the CCD chip and filter light. Owing to cost considerations, such packaging glass is predominantly crafted from quartz and similar materials, with the coating surface typically featuring anti-reflection coatings in the visible spectrum or incorporating a near-infrared filter. However, the transmittance of the mid-infrared band above 2.5 μm is not taken into account during coating. Consequently, the transmittance of mid-infrared laser radiation on the front part of the CCD was assessed via practical measurement methods. The packaging glass of the CCD was extracted, and a laser emitting at a center wavelength of 3.8 μm was employed to assess its transmittance (see Figure 7 and Figure 8). Utilizing a stage, the packaging glass was incrementally displaced, and the resultant alterations in energy meter readings were recorded to derive transmittance values. The collected data are presented in Table 3.

Examination revealed that the CCD’s packaging glass did not block the mid-infrared laser, exhibiting notable transmittance. Analysis of experimental data within this band revealed the laser’s damage threshold to the CCD lay in the millijoules range. Applying the heat conduction formula mentioned above, it was determined that millijoules-level energy density was necessary to impair the CCD’s microlens layer, which boasted the lowest melting point, while joules-level energy density was requisite for Si substrate damage. Considering the optical amplification of the medium-wave optical lens, laser output at the millijoule level satisfactorily fulfilled the experimental requisites.

### 4.2. Experiment on CCD Detector Damage by Mid-Infrared Pulsed Laser

**Figure 9:**
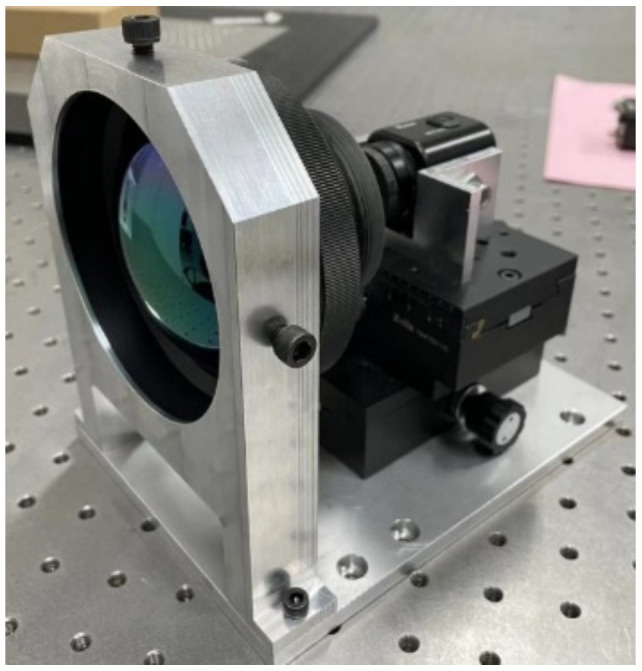
CCD camera with mid-IR lens attached.

The experimental setup remained consistent with the configuration outlined in Table 2. Customized tooling, depicted in Figure 9, facilitated the connection between the mid-infrared lens and the CCD detector. Mounted on the XY stage, the CCD detector underwent adjustment to align its focal plane with the back focal length of the lens, ensuring coaxial alignment between the CCD detector and the mid-infrared lens.

The experiment was conducted under identical experimental platforms and conditions as those of the in-band experiment. However, due to the 3.8 μm band lying beyond the response bandwidth of the CCD detector, discerning observation phenomena during the laser loading process proved challenging.

**Figure 10:**
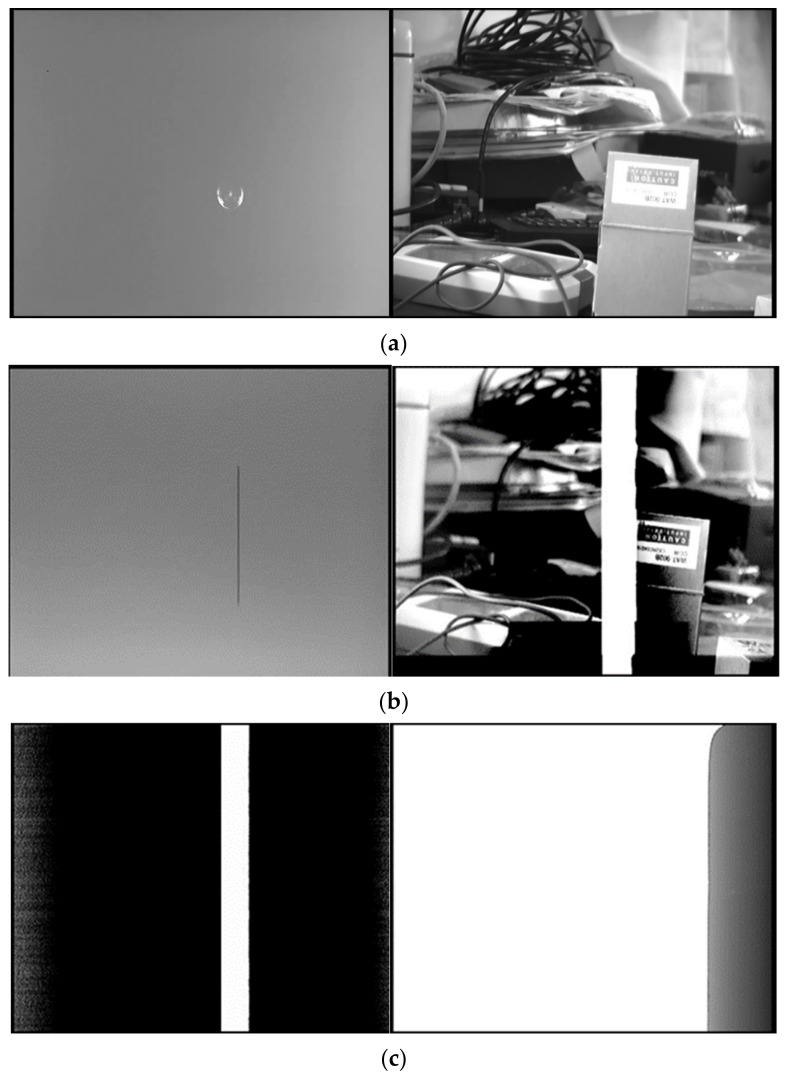
Experiment of mid-infrared pulse laser irradiation CCD detector. (**a**) Point damage. (**b**) Line damage. (**c**) Complete damage.

The experiment revealed a progressive deterioration in the CCD camera’s condition as the laser energy density increased, manifesting in sequential occurrences of point damage, line damage, and ultimately complete failure. Point damage commenced at an energy density of 0.883 J/cm^2^ under 1-on-1 conditions (Figure 10a), accompanied by the observation of a slight arc phenomenon by the CCD detector upon laser loading, indicative of the CCD’s responsiveness to mid-infrared laser radiation. Pixel-level dead pixels and short black thin lines appeared on the CCD. Notably, the threshold values for line damage and point damage were closely proximate, resulting in a subtle imaging effect. Subsequent escalation in laser energy led to line damage initiation at an energy density of 1.019 J/cm^2^ (Figure 10b), marked by the emergence of a black line during laser loading, followed by the appearance of a large area of band-shaped failure pixels post-loading, significantly impacting the image quality despite the CCD’s ability to capture images. Complete CCD failure ensued at an energy density of 1.892 J/cm^2^ (Figure 10c), characterized by a large-area saturation phenomenon upon loading and subsequent detection of comprehensive CCD malfunction. Material melting and short-circuiting of the base material were observed, resulting in irreparable detector damage.

## 5. Analysis of Damage Mechanism of Surface Array CCD by Mid-Infrared Pulse Laser

**Figure 11:**
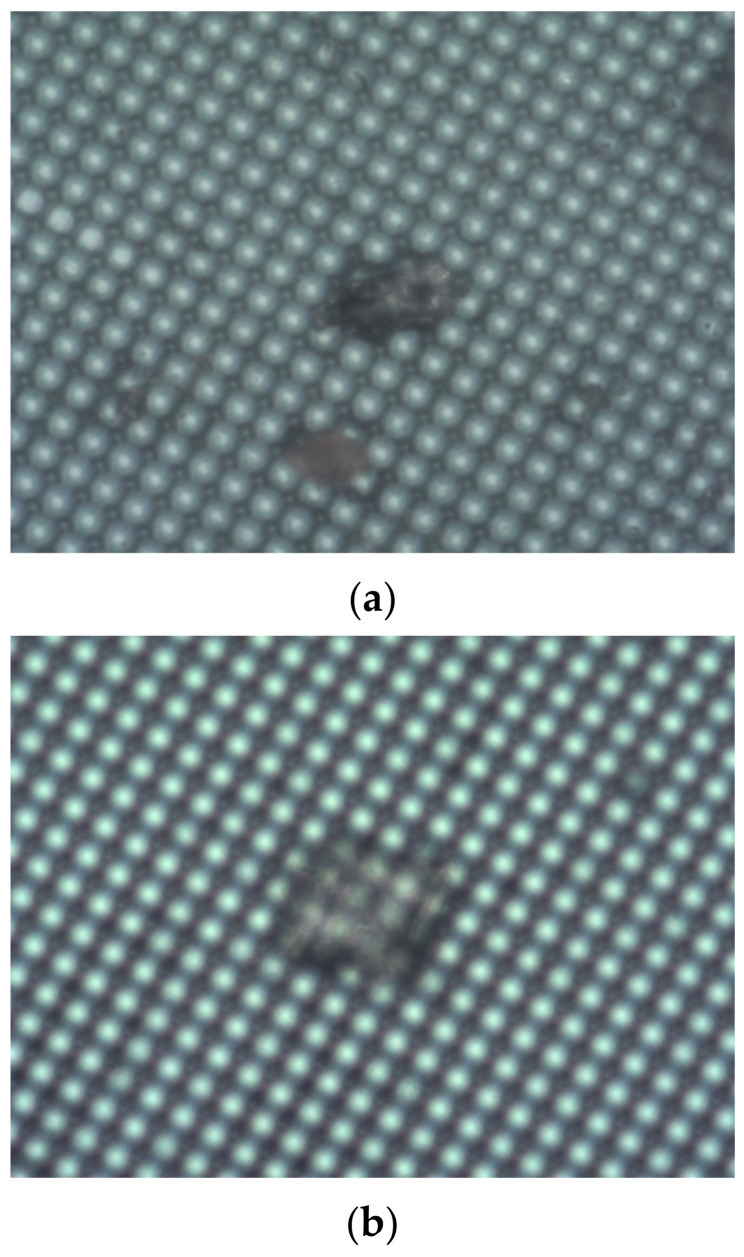

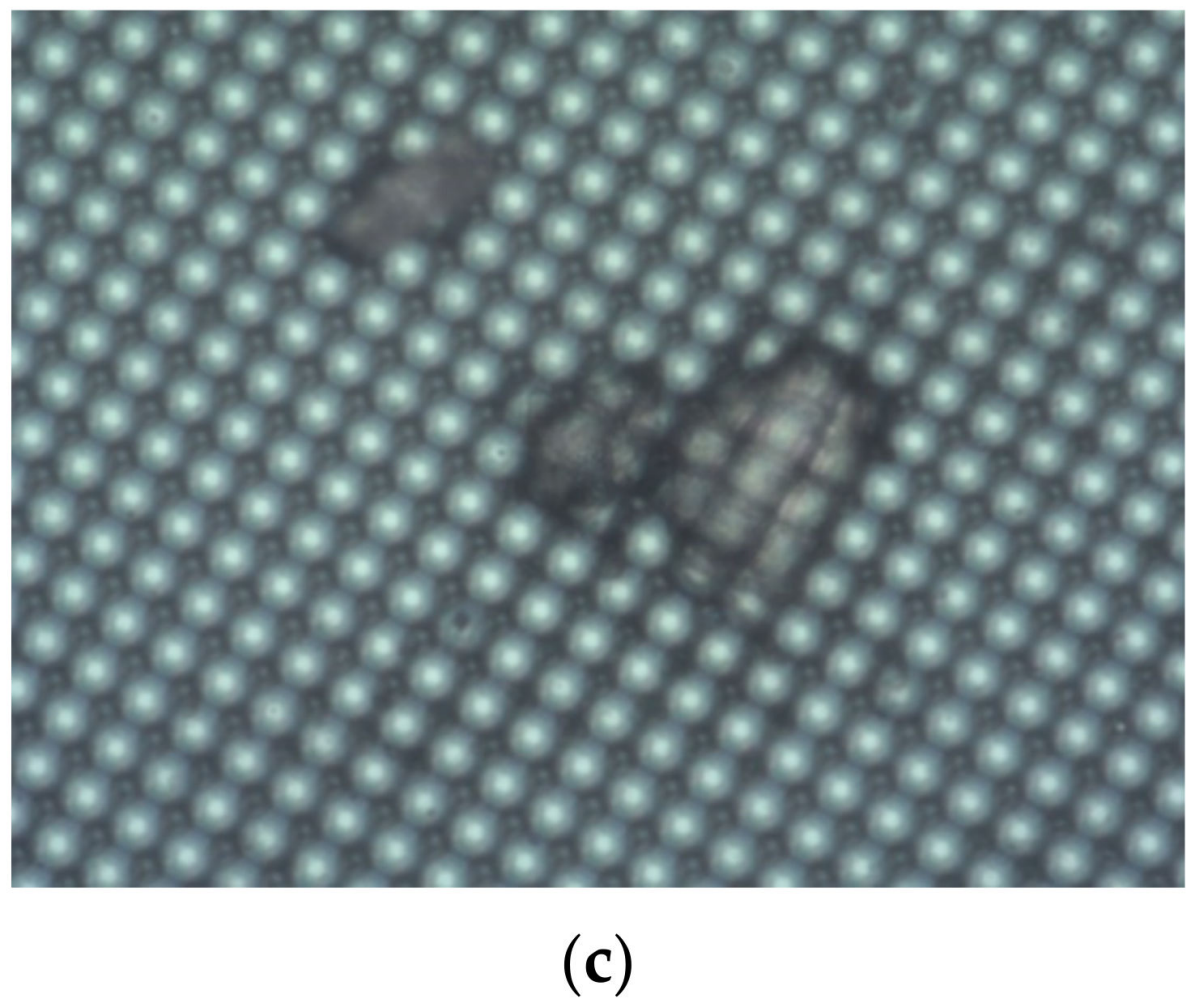
CCD surface topography. (**a**) Surface morphology of CCD in point damaged state. (**b**) Surface morphology of CCD in line damaged state. (**c**) Surface morphology of CCD in completely damaged state.

The analysis of the test outcomes revealed that out-of-band lasers with sufficient energy could drive the CCD into a state of saturation or supersaturation, albeit with imaging recovery possible upon cessation of laser loading. Evidently, causing irreversible damage to CCD detectors via out-of-band lasers posed challenges. Comparatively, CCD ablation induced by pulsed lasers was more straightforward. Once the depth of CCD destruction reached a critical threshold, its imaging capabilities were entirely compromised. However, shallow ablation depths resulted in localized damage, preserving the CCD’s overall imaging functionality. Examination of the CCD structure and damage morphology indicated that the microlens segment was the first to incur damage, undergoing pyrolysis upon reaching the damage threshold, consequently altering the laser’s focal point. Additionally, energy dissipation through thermal radiation and convection prevented materials from reaching their melting points and avoided destruction via melting. Stress-induced failure occurred when the silicon substrate attained material strength due to compressive stress, causing the bottom surface’s fixed edge to shift from external to internal during the initial laser irradiation stages. Given stress failure precedes thermal failure, detachment of the aluminum film may halt thermal failure or expedite stress failure. Thus, thermal damage emerged as the primary mechanism for mid-infrared laser-induced detector damage, culminating in the CCD detector’s complete failure due to chip short-circuiting. This damage trajectory corresponded to the observed point-line-blind damage depicted in Figure 11.

**Figure 12:**
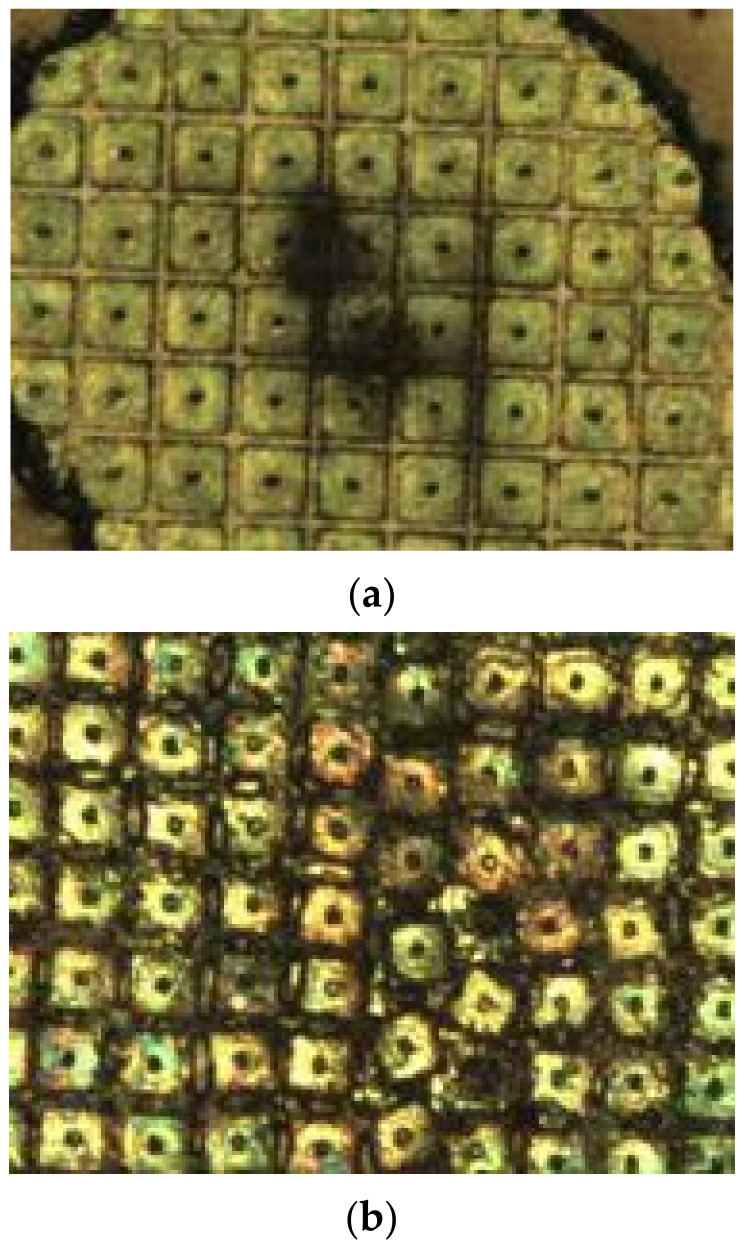
HgCdTe surface topography. (**a**) Surface morphology of the HgCdTe detector chip: filter film ablation. (**b**) Surface morphology of HgCdTe detector chip: pixel ablation.

The damage morphology of the mid-wave HgCdTe detector chip is shown in Figure 12. As can be seen from the bottom left image, a layer of filter film covered the top of the pixels. When the laser acted on the chip surface, there was significant ablation of the filter film, exposing the pixels. When the laser energy density was relatively low, there were no obvious changes in the pixels, and the ablation of the filter film left ablation marks on the pixel surface. As the laser energy increased, a larger area of the filter film was cleanly ablated, exposing more pixels to the laser-affected area. Figure 12b shows that the central circular groove of the pixels was the main location where the laser power was deposited and was also the first place where the pixels began to melt. As the irradiation time increased, the ablation area and depth gradually increased, causing some pixels to undergo thermal strain and displacement, altering their shape and position. For the vast majority of pixels, ablation at the photosensitive center was the direct cause of pixel damage.

The analysis of the damage threshold data presented in Table 4 and Table 5 revealed that the out-of-band damage threshold surpassed the in-band damage threshold, with the point, line, and complete damage thresholds being approximately 53%, 45%, and 49% higher, respectively. Notably, for mid-infrared pulsed lasers, the disparity between in-band and out-of-band damage thresholds approximated two times. Interestingly, the point damage threshold for both detectors closely approximated the line damage threshold, necessitating roughly two times the energy required for point damage to induce complete damage.

## 6. Discussion

1. The damage threshold of the mid-infrared pulsed laser to the CCD detector fell within the range of 0.883 J/cm^2^ to 1.892 J/cm^2^, whereas for the HgCdTe detector, it ranged from 0.412 J/cm^2^ to 0.953 J/cm^2^.
2. The mid-infrared pulsed laser exhibited a propensity for inducing permanent damage to CCDs, delineated into point damage, line damage, and complete blinding, each with varying impacts on the imaging fidelity. While point and line damage impaired specific regions’ imaging capability, the CCD’s overall functionality remained unaffected.
3. The damage mechanism of the mid-infrared pulsed laser on the CCD entailed thermal melting caused by the laser, with varying damage depths and sizes yielding distinct effects. Notably, the out-of-band damage threshold was approximately double that of the in-band damage threshold.

## Figures and Tables

**Table 1:** HgCdTe physical parameter from Bartoli.

Parameter	Value	Parameter	Value
Wavelength	3.8 μm	c	0.15 J·g^−1^K^−1^
k	0.09 cm^2^s^−1^	τ	150 ns
ΔT	916 K	α	2379 cm^−1^
R	0.31	a_0_	85 μm

**Table 2:** Experimental equipment.

Device	Device Parameters
HgCdTe detectors	Pixel size: 30 μm × 30 μm Resolution: 320 × 256 Frame rate: 100 FPS Integration time: 3 ms
Mid-IR lens	F: 2 Focal length: 100 mm Lens diameter: 50 mm
Mid-IR beam expander	Structure: Off-axis reflective Expansion: 10× Input beam diameter: 20 mm
DF laser	Center wavelength: 3.8 μm Maximum single pulse energy: 3.03 J Pulse width: 135 ns Maximum repetition rate: 50 Hz Initial divergence angle: ~10 mrad
Power meter 1	Spectral range: 0.19–20 μm Power range: 20 μW–2 W Energy range: 20 μJ–2 J
Power meter 2	Spectral range: 0.19–20 μm Power range: 150 mW–250 W Energy range: 80 mJ–10 J
Mid-IR attenuator	Structure: Reflective attenuator

**Table 3:** CCD package glass transmittance measurement results.

Laser Output Energy	Energy Detected by the Power Meter	Transmittance
170 mW	140.9 mW	82.9%

**Table 4:** Experimental data on damage to HgCdTe detectors by mid-infrared pulsed laser.

Laser Diameter	Laser Energy	Damage Phenomenon	Damage Threshold
210 μm	0.142 mJ	Point damage	0.412 J/cm^2^
210 μm	0.190 mJ	Line damage	0.551 J/cm^2^
210 μm	0.284 mJ	Complete damage	0.953 J/cm^2^

**Table 5:** Experimental data on damage to CCD detectors by mid-infrared pulsed laser.

Laser Diameter	Laser Energy	Damage Phenomenon	Damage Threshold
210 μm	0.304 mJ	Point damage	0.883 J/cm^2^
210 μm	0.353 mJ	Line damage	1.019 J/cm^2^
210 μm	0.655 mJ	Complete damage	1.892 J/cm^2^

## Data Availability

The data presented in this study are available upon request from the corresponding author.
